# STM2457 Inhibits the Invasion and Metastasis of Pancreatic Cancer by Down-Regulating BRAF-Activated Noncoding RNA N6-Methyladenosine Modification

**DOI:** 10.3390/cimb45110555

**Published:** 2023-11-03

**Authors:** Shaolong Hao, Haitao Sun, Hao Sun, Bo Zhang, Kailun Ji, Peng Liu, Fang Nie, Wei Han

**Affiliations:** 1 Department of General Surgery, Beijing Luhe Hospital, Capital Medical University, 82 Xinhua South Road, Tongzhou, Beijing 101149, China; haoshaolong2014@163.com (S.H.); 13260030347@163.com (H.S.); bianka2686@163.com (B.Z.); ji_kailun@163.com (K.J.); peterpengliu@163.com (P.L.); 2 Department of Central Laboratory, Beijing Luhe Hospital, Capital Medical University, 82 Xinhua South Road, Tongzhou, Beijing 101149, China; m18810253032@163.com (H.S.); fangnie2021@163.com (F.N.)

**Keywords:** pancreatic cancer, long noncoding RNA, N6-methyladenosine

## Abstract

Pancreatic cancer is a malignant tumor of the digestive system that is highly malignant, difficult to treat, and confers a poor prognosis for patients. BRAF-activated noncoding RNA (BANCR) has been proven to play an important role in the invasion and metastasis of pancreatic cancer. In this study, we focused on BANCR as a potential therapeutic target for human pancreatic cancer. The BANCR level in pancreatic cancer tissues and cells is affected by m6A methylation. Based on this, the aim of our study was to investigate the effect of a highly potent and selective first-in-class catalytic inhibitor of METTL3 (STM2457) on BANCR m6A methylation and its malignant biological behaviors in pancreatic cancer. The relationship between BANCR expression and BANCR m6A modification was detected with RT-qPCR and MeRIP-PCR. The expression of methyltransferase-like 3 (METTL3), the key enzyme involved in m6A methylation, in pancreatic cancer tissues was detected using a Western blot. STM2457 was used in vitro to investigate its resistance to the proliferation, invasion, and metastasis of pancreatic cancer cells. BANCR was overexpressed in pancreatic cancer tissues and cells, which was associated with poor clinical outcomes and validated in pancreatic cancer cell lines. m6A modification was highly enriched within BANCR and enhanced its expression. Remarkably, STM2457 inhibited the proliferation, invasion, and metastasis of pancreatic cancer cells by down-regulating BANCR m6A modifications. This study demonstrates the promise of BANCR as a new diagnostic and therapeutic target for pancreatic cancer and reveals the therapeutic effect that STM2457 exerts on pancreatic cancer by down-regulating BANCR m6A modifications.

## 1. Introduction

Pancreatic cancer (PC) is one of the most aggressive types of digestive system cancer and is the seventh leading cause of cancer-related deaths worldwide [1]. Pancreatic cancer has an extremely poor prognosis, with a 5-year survival rate of less than 8% [2]. Pancreatic cancer develops with variable symptoms, local invasiveness, or metastasis to distant sites in its early stages [3]. Although surgery, radiotherapy, and chemotherapy treatment have greatly improved over time, the prognosis of PC has not significantly improved in the past 20 years [4]. Recent advances revealed that only <2% of human genome transcripts code for proteins, and the remaining 98% of transcripts encode different classes of noncoding RNAs [5]. Though long noncoding RNAs (lncRNAs) comprise more than 200 nucleotides that lack protein-coding capacity, they are involved in a diverse range of biological processes, including cell growth, differentiation, and proliferation [6]. There is mounting evidence that lncRNAs play essential roles in PC cell proliferation, apoptosis, and metastasis. Accordingly, it is important to investigate the mechanisms through which lncRNA contributes to metastasis in pancreatic cancer.

RAF-activated non-protein-coding RNA (BANCR) is a lncRNA that is 693 bp in length and located in a gene desert region on 9q21.11-q21.12 [7]. In recent years, BANCR has been found to play a regulatory role in the proliferation, metastasis, and invasion of various malignant tumors such as gastric cancer [8], hepatocellular carcinoma [9], and thyroid adenocarcinoma [10]. In a previous study, we showed that BANCR is highly expressed in PC and could promote the progression of PC by regulating the HIF1a/VEGF-C/VEGFR-3 pathway [11]. In addition, Liu et al. [12] identified that BANCR is upregulated in pancreatic cancer tissues and cell lines, promotes pancreatic cancer tumorigenesis through the miR-195-5p/Wnt/β-catenin axis, and may serve as a potential target for diagnostics and therapeutics in pancreatic cancer. In this study, we found a high expression level of BANCR in pancreatic cancer tumor tissues and pancreatic cancer cell lines, and the expression of BANCR was positively correlated with lymph node metastasis in pancreatic cancer patients. It is suggested that BANCR may play a stimulative role in the development of pancreatic cancer. Further studies showed that pancreatic cancer cell proliferation, migration, and invasion function with BANCR knockdown were weakened. Although the important role of BANCR in PC invasion and metastasis has been identified, the specific mechanisms regulating BANCR levels require further exploration.

N6-methyladenosine (m6A) is the most abundant and well-known RNA epigenetic modification [13]. Studies have shown that more than 7000 different mRNAs and more than 300 lncRNA molecules have m6A methylation modification [14], suggesting that m6A methylation may widely affect gene expression. The installation of m6A modification is co-regulated by m6A methyltransferase (writers), demethylase (erasers), and some RNA-binding proteins (readers), and it is a dynamic and reversible process in the cell [15]. In this study, the MeRIP-PCR experiment showed that BANCR in pancreatic cancer cells had a high degree of m6A methylation. We speculated that m6A methylation was involved in regulating the expression of BANCR in PC. To investigate the therapeutic potential of targeting the m6A methylation of BANCR as an anti-PC strategy, we carried out experiments in vitro using the small molecule STM2457. STM2457 is a highly efficient, selective, and novel small-molecule inhibitor of RNA m6A methylation modification and has a highly specific inhibitory effect on the catalytic activity of METTL3. As a core factor in the m6A methylase complex, METTL3 plays the role of a “Writer” in the m6A methylation modification of various human malignant tumor regulators, which can affect the processing of target RNA by promoting the m6A level of target genes and recruiting specific “readers” [16]. In recent years, METTL3 has been identified as a carcinogenic driving factor of PC [17,18]. A highlight of the work by Yankova, Blackaby et al. was their reporting of the inhibitory effect of STM2457 on mediated m6A modification; they showed safe and effective anticancer effects in both in vivo and in vitro leukemia models [19], revealing the potential value of STM2457 as a target for RNA m6A inhibition of malignant tumors. This study attempted to explore the therapeutic effect of STM2457 targeting BANCR m6A on pancreatic cancer proliferation, migration, and invasion. This study further expands the function of m6A methylation to modify BANCR and provides new ideas for the prevention and treatment of pancreatic cancer invasion and metastasis.

## 2. Materials and Methods

### 2.1. Cell Lines and Chemicals

Cell lines PANC-1, SW1990, and immortalized human pancreatic ductal epithelial cells (HPDECs) were purchased from Guangzhou Genio Biotech Co., Ltd. (Guangzhou, China). The SW1990 and HPDEC cells were cultured in DMEM (Sigma-Aldrich, Shanghai, China), and the PANC1 cells were cultured in RPMI1640 (HyClone, Logan, UT, USA), each supplemented with 10% FBS (Thermo Fisher Scientific, Waltham, MA, U Logan SA) and 1% antibiotics (Thermo Fisher Scientific, Waltham, MA, USA). Culture was performed under the following conditions: 37 °C, 20% O_2_, 5% CO_2_, and 75% N_2_. Cells in the logarithmic growth stage were selected for subsequent experiments.

### 2.2. Real-Time qRT-PCR

The mRNA expression levels of genes were determined using real-time qRT-PCR as described previously [20]. A TRIzol^®^ RNA extraction kit was used to extract total cell RNAs according to the manufacturer’s instructions. The relative expression levels of BANCR in each group were detected by RT-qPCR. RT-qPCR was performed in strict accordance with the instructions of the SYBR^®^ Prime Script™ RT-PCR Kit (Takara Bio, Beijing, China), with GAPDH as the internal reference gene in a reaction system of 20 μL. The reaction conditions were as follows: Pre-denaturation at 95 °C for 5 min, followed by 38 cycles of denaturation at 95 °C for 30 s, annealing at 65 °C for 30 s, and extension at 72 °C for 30 s; and a final extension step of 72 °C for 8 min. Primer sequences are shown in Supplementary Materials Table S1.

### 2.3. Western Blotting

A BCA assay was used to determine the protein concentration, and gel electrophoresis was performed using 20 μg of samples per lane. Electrophoresis was performed at a constant voltage of 100 V for 40 min. After electrophoresis, the electroporation apparatus was used to transfer the resolved proteins to a membrane. Membrane transfer was accomplished with a constant current of 250 mA for 2 h. The proteins were transferred to PVDF membranes, which were subsequently blocked at room temperature for 1 h with 5% skimmed milk/TBST solution. The dilution of the primary antibody against METTL3 was 1:500. A GAPDH antibody (ZSJQ, Beijing, China) was selected as the internal reference and incubated overnight at 4 °C. After washing the film with TBST three times, the film was incubated with the secondary antibody (Beijing Zhongshan Jinqiao) at room temperature for 1 h. TBST was used to wash the films again three times, after which the signals were developed and visualized using ECL reagent (Thermo Fisher Scientific, Waltham, MA, USA). A Canoscan Lide 120 scanner was used to analyze the film for densitometry. Densitometry analysis was performed using ImageJ 1.48.

### 2.4. Immunofluorescence In Situ Hybridization

PC cells were seeded on glass coverslips overnight and treated with STM2457 for 24 h. The treated cells were fixed and incubated with a primary antibody, followed by incubation with a secondary antibody. Coverslips were then mounted with an anti-fade mounting solution supplemented with DAPI. The images were then captured using a confocal microscope (Leica Microsystems, Wetzlar, Germany).

### 2.5. RNA Isolation and Quantitative Real-Time PCR

According to the manufacturer’s instructions, total RNA was extracted from cells or tissues using AipPure TRIzol Total RNA Extraction Reagent (i-presci, Beijing, China). For miRNA assays, TaqMan MicroRNA assays (Applied Biosystems; Thermo Fisher Scientific, Waltham, MA, USA) were used to quantify the expression levels of mature miRNAs. cDNA was synthesized from total RNA using a TaqMan MicroRNA Reverse Transcription Kit (Thermo Fisher Scientific, Waltham, MA, USA) with assay-specific TaqMan primers, and quantitative real-time PCR was performed using 2× TaqMan Universal PCR Master Mix (Thermo Fisher Scientific, Waltham, MA, USA). The following assay IDs were used: BANCR (ncRNA, 100885775), METTL3 (protein-coding, 56339), and GAPDH (protein-coding, 2597) (all purchased from Applied Biosystems; Thermo Fisher Scientific, Waltham, MA, USA). The reactions were performed on a 7500 Real-Time PCR System (Thermo Fisher Scientific, Waltham, MA, USA). Relative expression levels were calculated using the 2^−∆∆Ct^ method.

### 2.6. MeRIP-qPCR

MeRIP assays were conducted using the EpiQuik TM CUT&RUN m6A RNA Enrichment (MeRIP) kit (Epigentek, Farmingdale, NY, USA), following the manufacturer’s instructions. First, RNA was extracted from PC cells or tissues and fragmented via sonication at 0 °C for 20 s, after which magnetic beads (Sigma-Aldrich, Shanghai, China) were incubated at room temperature for 90 min with anti-m6A. Antibody-conjugated beads were then rinsed, combined with DNA-free RNA for 15 min at 4 °C with constant rotation, and then washed again. RNA was then eluted with an eluted buffer (m6A). A miRNeasy mini-kit (QIAGEN, Dusseldorf, Germany) was used to isolate this RNA, after which qPCR analyses were conducted as appropriate.

### 2.7. Cell Proliferation Assays 

Cell Counting Kit-8 (CCK-8) (EnoGeneCell, Nanjing, China) was used as the manufacturer’s protocol. After treatment, the CCK-8 solution was added, and the absorbance at 450 nm was recorded using a microplate reader (Thermo Fisher Scientific, Waltham, MA, USA).

### 2.8. Colony Formation Assay

Each group of cells, in their exponential growth phase, was seeded into a six-well plate at 1000 cells per well, and then 2 mL of 10% FBS-supplemented DMEM medium was added to each well, mixed, and incubated for 7 to 14 days. Later, it was fixed with 4% paraformaldehyde and stained with 0.5% crystal violet. 

### 2.9. Wound Healing Assay 

After treatment, the cells were scratched with pipette tips. Images were taken at the indicated time with an optimal microscope and analyzed using ImageJ 1.52K software. For CAL27, the indicated time was 30 h, while for WSUHN6, it was 8 h.

### 2.10. Cell Migration and Invasion Assay

Transwell assays were conducted with an 8-μm pore size insert with or without Matrigel Invasion Chambers to evaluate the properties of migration or invasion of pancreatic cells. Then, 48 h post-transfection, single-cell suspensions were seeded into the upper chamber, and the lower chamber was filled with 500 μL of DMEM containing 10% FBS. After the indicated incubation time, the non-migrating or invading cells were wiped from the upper surface of the membranes, and cells were stained with a 2% crystal violet solution after fixation in methanol. Images of migrated cells in each well were captured and counted in random fields using a microscope. Each experiment was conducted in triplicate.

### 2.11. Statistical Analysis

The data were analyzed using the Prism software program (version 9) (Graph Pad Software, San Diego, CA, USA). Data were expressed as mean ± SEM. Statistical analyses between control and treatment groups were performed using a student’s *t*-test. *p* < 0.05 was considered to be statistically significant.

## 3. Results

### 3.1. BANCR Was Overexpressed in Pancreatic Cancer Tissues and Cells, Which Are Associated with Poor Clinical Outcomes

**Figure 1:**
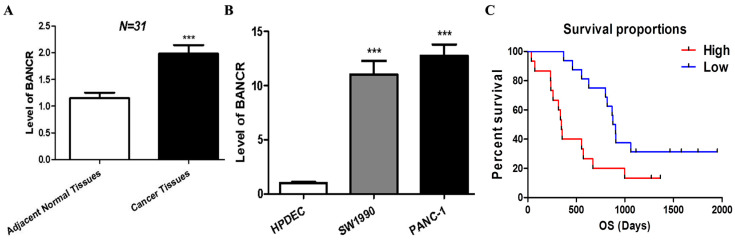
BANCR was upregulated in pancreatic cancer tissues and cell lines, which are associated with poor clinical outcomes. (**A**) Bar graph representation of the differences in levels of BANCR in PC tumor tissues and adjacent normal pancreatic tissues. ***, *p* < 0.001. (**B**) Bar graph representation of the changes in levels of BANCR in PC cell lines (SW1990 and PANC-1 cells). ***, *p* < 0.001. (**C**) Kaplan–Meier plots and *p*-values of the log-rank test for comparing the survivals of PC patients with high and low expression of BANCR.

The level of BANCR in 31 pancreatic cancer tissues and paired adjacent normal control tissues was detected by RT-qPCR. As shown in Figure 1A, the level of BANCR in 77.4% (24/31) of pancreatic cancer tissues (2.45 ± 0.18) was higher than that in the adjacent normal tissues (1.31 ± 0.12). The expression of BANCR in pancreatic cancer cell lines SW1990 (11.04 ± 1.26) and PANC-1 (12.77 ± 1.03) was higher than normal pancreatic ductal epithelium cells (HPDECs) (Figure 1B). Therefore, it was decided to knock down BANCR in PANC-1 cells for further study. We analyzed the correlations between the expression levels of BANCR in PC tumor tissues and patients’ clinicopathological parameters. As shown in Table 1, samples of 31 patients were collected, with an average age of 66.06 ± 8.37 years old (43–79 years old). All patients were diagnosed with pancreatic ductal adenocarcinoma by pathological specimens after surgery, and the postoperative follow-up time was about 3 years. SPSS22.0 statistical software was used for single-factor correlation analysis of clinicopathological data, and the results showed that the relative expression of BANCR was significantly correlated with the patient’s age, lymph node metastasis, histological grade, and advanced clinical stage (*p* < 0.05). In addition, we assessed the survival durations of the 31 PC patients corresponding to these obtained samples. A Kaplan–Meier analysis showed that patients with a high expression of BANCR in PC tissues had worse survival rates than those with low expression (HR = 2.942, 95% CI 1.226–7.061, *p* = 0.015). (Figure 1C). These results demonstrate an increased BANCR in PC tumor specimens and suggest that it may help predict the aggressiveness of PC patients.

### 3.2. BANCR Promotes Pancreatic Cancer Cell Proliferation, Migration, and Invasion

**Figure 2:**
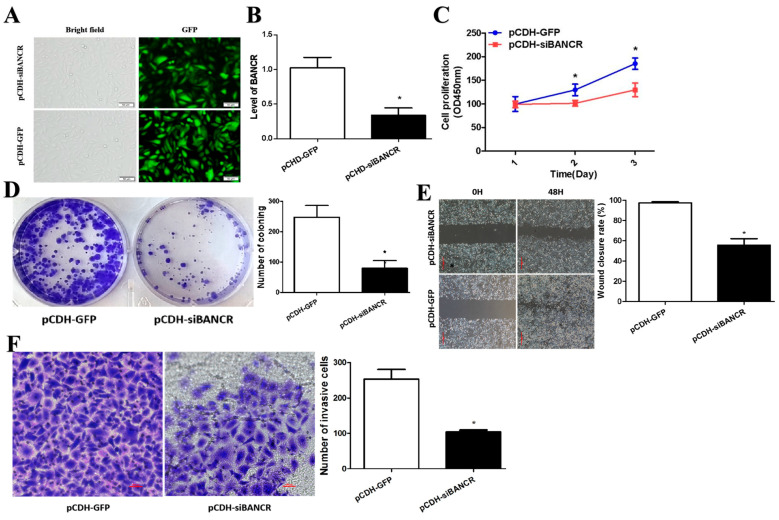
Knocking down BANCR inhibits pancreatic cancer cell proliferation, migration, and invasion. (**A**) Inverted fluorescence microscopy to identify PANC-1 cell lines transfected with knockdown BANCR (is-BANCR) and no-load control (GFP) (GFP: Green Fluorescent Protein, ×200). (**B**) RT-qPCR to verify successful knockdown of BANCR in PANC-1 cells. (**C**,**D**) CCK-8 assay. *, *p* < 0.05. (**C**) and colony formation assay. *, *p* < 0.05. (**D**) were used to determine the viability of PANC-1 cells in well of 6-well plates after knocking down BANCR. *, *p* < 0.05. (**E**,**F**) Wound healing assay (**E**) and Transwell invasion assay (Scale bars, 500 µm) (**F**) were performed to evaluate the migration and invasion ability of PANC-1 cells after knocking down BANCR (Scale bars, 100 µm). *, *p* < 0.05.

As shown in Figure 2A,B, the expression of BANCR in the established pancreatic cancer stable cell lines PANC-1 is significantly different. It is suggested that BANCR experiences low expression in PANC-1 cell lines of knockdown BANCR (pCDH si-BANCR), which can be used for further experiments. As shown in Figure 2C, the effect of BANCR on the proliferation of pancreatic cancer cells was tested by the CCK8 assay. The results showed that the proliferation of PANC-1 cells with low expression of BANCR significantly decreased compared with no-load pCDH (pCHD-GFP). It is suggested that low expression of BANCR can reduce the proliferation ability of pancreatic cancer cells. The plate cloning experiment is shown in Figure 2D. The influence of BANCR on the proliferation of pancreatic cancer cells was further detected by the plate cloning experiment. The results showed that the number of clones of PANC-1 cells of BANCR was significantly reduced compared with no-load pCDH. Therefore, it is suggested that the low expression of BANCR can reduce the proliferation ability of pancreatic cancer cells. Cell lines of knockdown BANCR inhibit the migration and invasion of pancreatic cancer cells, as shown in Figure 2E. A Transwell experiment was conducted to observe the effect of BANCR on the migration and invasion ability of pancreatic cancer cells. The results showed that the number of migration and invasion cells of PANC-1 cell lines knockdown BANCR was significantly reduced compared with empty pCDH cells (Figure 2F). These results indicate that BANCR can promote the migration and invasion abilities of pancreatic cancer cells.

### 3.3. m6A Modification Was Associated with the Up-Regulation of BANCR in PC Tissues and Cells

**Figure 3:**
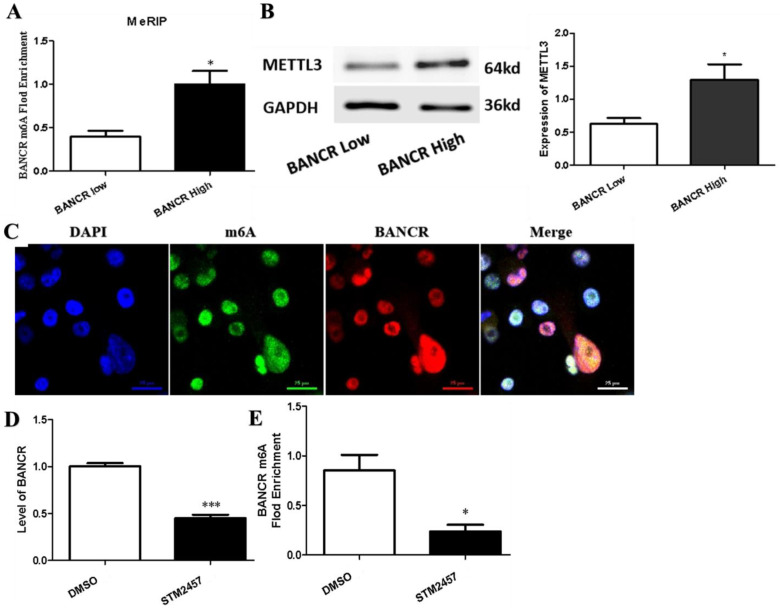
The m6A modification was enriched in BANCR, and it upregulated its transcription level. (**A**) MeRIP-qPCR determined m6A methylation levels of BANCR in BANCR low- and high-expression PC tumor tissues. *, *p* < 0.05. (**B**) Western blot analysis comparing the differences in expression of METTL3 in BANCR low- and high-expression PC tumor tissues. *, *p* < 0.05. (**C**) Immunofluorescence analyzed the localization and expression of m6A (green), BANCR (red) and nucleus (blue) in PANC-1 cells, and nuclei were stained with DAPI. (**D**,**E**) Identification of STM2457 affects the level of BANCR (**D**) and its’ m6A modification (**E**) in PANC-1 cells. *, *p* < 0.05; ***, *p* < 0.001.

m6A modification is the most prevalent post-transcriptional modification of lncRNA, and it has both translation and stability. In order to explore the underlying mechanism by which BANCR is upregulated in PC, the relationship between BANCR expression and BANCR m6A modification in PC tumor tissues was detected by RT-qPCR and MeRIP-PCR. As shown in Figure 3A, the level of BANCR m6A was higher in PC tumor tissues with highly expressed BANCR compared to lowly expressed BANCR. METTL3 plays a core role in the m6A methylation modification of PC. RT-qPCR was performed to observe the relationship between the expression of METTL3 and BANCR. We found that METTL3 was significantly positively correlated with BANCR in PC tissues (Figure 3B). As shown in Figure 3C, immunofluorescence in situ hybridization showed that BANCR and m6A were located in the nucleus of the cell, indicating that m6A methylation occurred after transcription of BANCR. Scanning with a laser confocal microscope, red represents BANCR, green represents m6A, and yellow represents Merge. It has been confirmed above that METTL3 is related to the levels of BANCR. This study will further explore the effects of STM2457, a small-molecule inhibitor of METTL3, on the BANCR expression and m6A methylation of pancreatic cancer cells. We performed a Western blot analysis and RT-qPCR to detect the expression of METTL3 and BANCR in PANC-1 cells after STM2457 treatment using DMSO treatment as the control. As shown in Figure 3D, STM2457 significantly reduced the expression of METTL3 and BANCR. Furthermore, the MeRIP-seq indicated a significant reduction in BANCR m6A modification after STM2457 was treated in PANC-1 cells (Figure 3E).

### 3.4. STM2457 Exerts Anticancer Activities in Pancreatic Cancer Cells In Vitro

**Figure 4:**
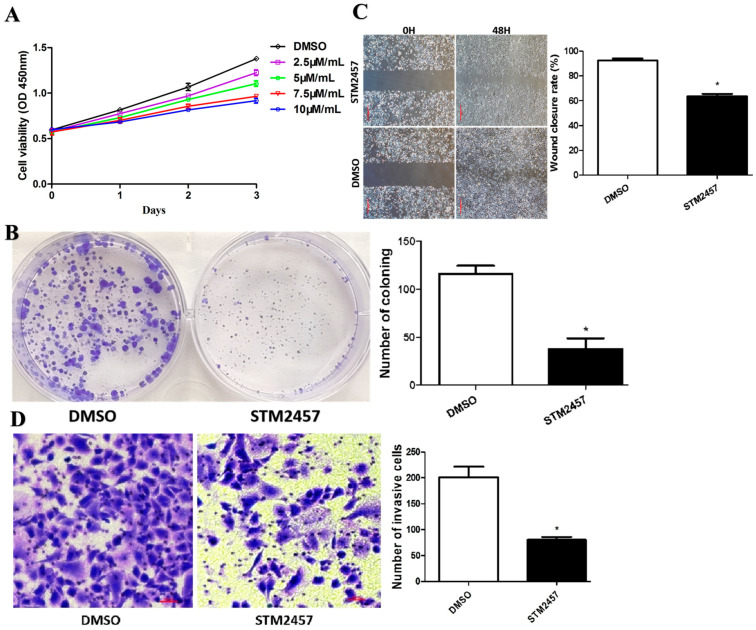
STM2457 exerts anticancer activities in pancreatic cancer cells in vitro. CCK-8 assay (**A**) and colony formation assay (**B**) were used to determine the viability of PANC-1 cells after treatment between vehicle (DMSO) and STM2457 in well of 6-well plates. Wound healing assay (**C**) and transwell invasion assay (Scale bars, 500 µm) (**D**) were performed to evaluate the migration and invasion ability of PANC-1 cells post-treatment between vehicle (DMSO) and STM2457 (Scale bars, 100 µm). *, *p* < 0.05.

In order to observe the effect of STM2457 on the proliferation, migration, and invasion ability of pancreatic cancer cells, PANC-1 cell lines were treated with different concentrations of STM2457 for 72 h. As shown in Figure 4A, the effect of STM2457 on the proliferation of pancreatic cancer cells was tested using the CCK8 assay and colony formation. The results showed that the proliferation of lowly expressed PANC-1 cells with BANCR significantly decreased compared with pCHD-GFP (Figure 4A,B). We further performed wound healing assays and Transwell invasion assays to assess the effects of STM2457 on pancreatic cancer cell migration and invasion, respectively. As shown in Figure 4C, the cell wound healing assay experiment showed that PANC-1 cells treated with STM2457 significantly reduced the healing rate compared with DMSO, with a statistical difference. The Transwell invasion experiment showed that PANC-1 and SW1990 cell lines treatment with STM2457 significantly reduced the number of invasion cells compared with DMSO with a statistical difference (Figure 4D). Therefore, the above results indicated that STM2457 could inhibit the proliferation, migration, and invasion abilities of pancreatic cancer cells.

## 4. Discussion

There is an urgent need to develop novel therapeutic targets for pancreatic cancer [21]. Long noncoding RNA (lncRNA) is abnormally expressed in a variety of human malignant tumors and can directly or indirectly participate in the processes of tumor proliferation, invasion, and metastasis [22]. In this study, we have focused on BANCR as a potential therapeutic target for human pancreatic cancer. In fact, there is increasing evidence that BANCR is overexpressed in human cancers, such as esophageal squamous cell carcinoma [23] and breast cancer [24]. BANCR is also upregulated in colorectal cancer plasma samples [25], and BANCR knockdown suppressed colorectal cancer progression and strengthened the chemosensitization of colorectal cancer cells to adriamycin [26]. As a malignant tumor-related lncRNA, BANCR regulates multiple signaling pathways through multiple links and plays a variety of roles in tumor cell proliferation, survival, migration, tumorigenesis, and metastasis [27]. Inhibiting this pathway could be a treatment option. Here, we found that BANCR m6A methylation increased in pancreatic tumor tissues and cells, upregulated BANCR level overexpression, and upregulated BANCR predicted poor prognosis in pancreatic cancer patients. The knockdown BANCR inhibited pancreatic cancer cell proliferation, migration, and invasion. This suggests that BANCR is an oncogene that promotes a poor prognosis in pancreatic cancer cells. Our experimental data suggest that modification by inhibiting m6A methylation of BANCR may be a promising biomarker and therapeutic target for the treatment of pancreatic cancer patients.

Among the various modifications, m6A is the most common and effective modification of coding RNA and noncoding RNA [28]. In recent years, a large number of targeted drugs have been developed for m6A modification, and some small molecules are under clinical study. However, these small molecules have been observed to have limited or no effect on pancreatic cancer, leading to the exploration of new targets and using them as a basis to develop new drugs that can affect BANCR levels. The BANCR level in pancreatic cancer tissues and cells is affected by m6A methylation. Based on this, we investigated the effect of STM2457 on BANCR m6A methylation and its malignant biological behaviors in PC. As a novel small-molecule m6A methylase inhibitor, STM2457 has been identified in vivo and in vitro to effectively inhibit disease progression in AML mouse models without causing significant host toxicity, but its role in pancreatic cancer remains unclear. This study showed that STM2457 can significantly down-regulate the methylation and expression levels of BANCRm6A and effectively inhibit the proliferation, migration, and invasion of pancreatic cancer cells in vitro. Therefore, STM2457 could be a targeted drug for pancreatic cancer treatment. Our work marks the first attempt to demonstrate that STM2457 targeting BANCRm6A may be an alternative strategy for controlling pancreatic cancer progression. However, the molecular mechanism by which STM2457 inhibits BANCRm6A is unexplored, which is the goal of our next work. In addition, STM2457 may play an anticancer role in BANCR and other types of cancers with high levels of m6A, and its efficacy and safety should be evaluated in more clinically relevant cancer models in the future.

### Limitations

Despite the significant results of the current study, some shortcomings still need to be considered. The present study was a single-center retrospective study with a small sample size, and therefore, further studies with larger sample sizes should be conducted. In pancreatic cancer, the molecular mechanism of BANCRm6A inhibition by STM2457 needs to be further explored [29,30,31]. The inhibitory effect of STM2457 on pancreatic cancer has not been verified in experimental animals.

## 5. Conclusions

In this study, we confirmed the clinical correlation between BANCR and pancreatic cancer and investigated the effect of STM2457 on the modification and expression of BANCR m6A at in vitro levels. This inhibitor showed an anticancer effect in the in vitro pancreatic cancer cell model that was mediated by affecting the expression of BANCR. In conclusion, our study demonstrates the potential of BANCR as a promising new diagnostic and therapeutic target for pancreatic cancer and reveals the therapeutic effect of STM2457 on pancreatic cancer mediated by inhibiting BANCR expression. In the future, we must investigate the molecular mechanism by which STM2457 affects BANCRm6A further and find the structural location of the interaction to lay the foundation for STM2457 to be used in clinical treatment.

## Figures and Tables

**Table 1:** Correlation between clinical parameters and the level of BANCR in PC tissues.

Parameter	No. of Patients (n = 31)	Level of BANCR (Mean ± SD)	*p* Value
Sex			
Male	21	2.135 ± 1.012	0.174
Female	10	1.663 ± 0.478	
Age (years)			
>65	18	2.252 ± 0.903	0.046 *
≤65	13	1.609 ± 0.766	
Tumor Size (cm)			
T1	4	1.033 ± 0.240	0.057
T2	11	1.867 ± 0.770	
T3	8	2.460 ± 1.180	
T4	8	2.138 ± 0.587	
Lymph Node Metastasis			
N0	11	1.445 ± 0.594	<0.001 ***
N1	16	2.010 ± 0.653	
N2	4	3.351 ± 1.072	
Distant Metastasis			
Yeas	3	2.578 ± 1.330	0.231
No	28	1.919 ± 0.846	
Histological Grade			
Well	9	1.656 ± 0.923	0.036 *
Moderate	15	1.757 ± 0.763	
Poor	7	2.613 ± 0.842	
Clinical Stage			
I	9	1.339 ± 0.503	0.024 *
II	9	1.915 ± 0.759	
III	10	2.444 ± 0.876	
IV	3	2.578 ± 1.330	

*, *p* < 0.05; ***, *p* < 0.001.

## Data Availability

All data and materials are available in the main text.

## Supplementary Materials

The following supporting information can be downloaded at: https://www.mdpi.com/article/10.3390/cimb45110555/s1, Table S1: The primer sequence table.
