# Supplementary material for: STM2457 Inhibits the Invasion and Metastasis of Pancreatic Cancer by Down-Regulating BRAF-Activated Noncoding RNA N6-Methyladenosine Modification
Source: Curr Issues Mol Biol. 2023 Nov 3;45(11):8852–63. doi: 10.3390/cimb45110555 (PMC10670688; doi:10.3390/cimb45110555)
Supplement: Supplementary file 1 [file cimb-45-00555-s001.zip › cimb-2634242-supplementary.pdf]

Table S1. The primer sequence table

Gene name		5’-3’Sequence
**GAPDH**	Sense	TCAAGAAGGTGGTGAAGCAGG
	Antisense	TCAAAGGTGGAGGAGTGGGT
**BANCR**	Sense	GAGCCTTGCCAGTTCCATTT
	Antisense	TGCAGAGGTGAGATTCAGGT
